# Spatiotemporal functions of leukemia inhibitory factor in embryo attachment and implantation chamber formation

**DOI:** 10.1038/s41420-024-02228-4

**Published:** 2024-11-25

**Authors:** Shizu Aikawa, Takehiro Hiraoka, Mitsunori Matsuo, Yamato Fukui, Hidetoshi Fujita, Tomoko Saito-Fujita, Ryoko Shimizu-Hirota, Norihiko Takeda, Daiki Hiratsuka, Xueting He, Chihiro Ishizawa, Rei Iida, Shun Akaeda, Miyuki Harada, Osamu Wada-Hiraike, Masahito Ikawa, Yutaka Osuga, Yasushi Hirota

**Affiliations:** 1 Department of Obstetrics and Gynecology, Graduate School of Medicine, The University of Tokyo, Tokyo, Japan; 2 Research Institute for Microbial Diseases, Osaka University, Suita, Osaka, Japan; 3 Department of Biomedical Engineering, Osaka Institute of Technology, Osaka, Japan; 4 Division of Cancer Biology, The Cancer Institute of Japanese Foundation for Cancer Research, Tokyo, Japan; 5 Department of Internal Medicine, Center for Preventive Medicine, Keio University School of Medicine, Tokyo, Japan; 6 Department of Cardiovascular Medicine, Graduate School of Medicine, The University of Tokyo, Tokyo, Japan

**Keywords:** Developmental biology, Reproductive biology, Infertility

## Abstract

Embryo implantation is crucial for successful pregnancy, requiring appropriate uterine responses to implantation-competent blastocysts. Molecular communication at the maternal–fetal junction governs this process. Leukemia inhibitory factor (Lif) plays a pivotal role in implantation across species. Lif is abundantly expressed in the glandular epithelium during blastocyst-receptive phase and is induced in the stroma surrounding attached blastocysts. While diminished Lif expression leads to infertility, its influence on peri-implantation uteri remains unclear. Therefore, we investigated the role of Lif in uterine physiology using its uterine-specific knockout (uKO) and uterine epithelial-specific KO (eKO) in mice. *Lif* eKO and uKO mice displayed infertility owing to failed embryo attachment. Recombinant Lif supplementation rescued the reproductive phenotype of *Lif* eKO mice, but not *Lif* uKO mice; however, recombinant Lif injection rescued embryo attachment in *Lif* uKO mice. RNA-seq analysis indicated that Lif governs uterine epithelial genes, but not embryonic genes, to facilitate embryo attachment via activating nuclear Stat3. Concordantly, three-dimensional imaging of the uterine epithelium revealed that luminal closure and crypt formation are regulated by the uterine Lif–Stat3 axis as well as the presence of blastocysts. Collectively, our findings shed light on previously unknown mechanism on how Lif influences uterine functions molecularly and physiologically during early pregnancy.

## Introduction

Infertility is a severe problem encountered by ~15% of couples worldwide [1]. While artificial reproductive technology (ART) have greatly advanced in the last decades, their successful rates remain ~30% [2]. Embryo implantation is a crucial step that influences subsequent decidualization, placentation, and pregnancy maintenance. Consequently, aberrant embryo implantation is a major cause of pregnancy failure [2–4]. Implantation failure can result in abortion, preterm birth, and mortality of newborn pups in mice [2, 3]. Additionally, the endometrium becomes receptive to implantation-competent blastocysts in the morning on pregnancy day 4 (day 1 = plug-positive day). After blastocyst attachment to the luminal epithelium on midnight of day 4, implantation chambers become evident and are accompanied by well-extended glands [5, 6]. Concurrently, the initiation of decidualization in the surrounding stroma becomes apparent.

Leukemia inhibitory factor (Lif) shares biological similarities with interleukin-6, functioning as a cytokine [7]. Lif activates its receptors, Lifr and gp130, initiating downstream Jak/Stat signaling. The pivotal role of Lif in ensuring successful pregnancy is widely recognized. In mice, Lif is highly expressed in the glandular epithelium during the receptive phase and subsequently in the stroma surrounding attached blastocysts [8, 9]. Systemic deletion of *Lif* in female mice results in complete infertility, likely owing to aberrant embryo implantation [9–11]. Earlier investigations, including our own, indicated compromised embryo implantation in mice with uterine-specific knockout (KO) of *Lifr*, *Gp130*, and *Stat3*, which are putative downstream effectors of Lif in uterine contexts [12–16]. Correspondingly, diminished uterine Lif levels correlate with infertile phenotypes across diverse mouse models [17–20]. Accordingly, Lif is likely crucial in human pregnancy; the Lif–Lif receptor (Lifr and gp130) axis is downregulated in infertile endometria during the secretory phase [21]. However, the precise mechanisms by which epithelial and stromal Lif coordinate endometrial readiness for embryo implantation remain unexplored.

Accordingly, we established conditional KO mice of *Lif* in the uterine epithelium (*Lif* eKO) and the entire uterus (*Lif* uKO) using *Ltf-iCre* and *Pgr-Cre* drivers, respectively, to investigate the role of Lif on the endometrium and attached blastocysts during embryo implantation. Overall, we aimed to elucidate previously unexplored mechanisms by which Lif orchestrates successful embryo implantation by facilitating embryo-chamber formation, thereby aiding in the development of novel approaches to address infertility and enhance ART.

## Results

### *Lif* eKO and uKO cause infertility

**Fig. 1:**
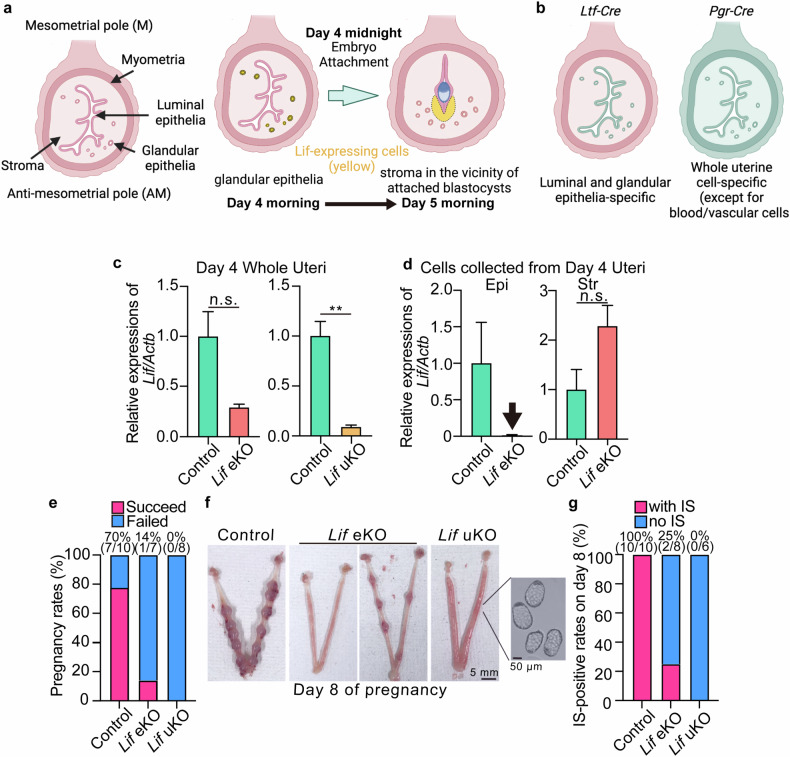
Uterine deletion of *Lif* results in severe infertility. **a** Schematic diagram of the spatiotemporal expression patterns of Lif. Lif is expressed in the uterine glands during the receptive phase and then in the stroma surrounding attached embryos. **b** Schematic diagram of gene deletion sites induced by *Ltf*-Cre (left) and *Pgr-*Cre (right) in mouse uteri. **c** *Lif* expression in whole uterine tissues from *Lif* eKO mice (left) and *Lif* uKO mice (right) on day 4 of pregnancy. *n* = 3 per genotype, n.s.: not significant, **P < 0.01 (Student’s *t*-test). **d** Epithelial-specific deletion of *Lif* was confirmed in epithelia and stroma isolated from *Lif* eKO uteri on day 4. *n* = 4 per genotype. **e** Severe infertility was confirmed via reduced pregnancy rates in mice of each genotype. Percentages of pregnancy rates and numbers of female mice with successful delivery/total tested are presented above the bars. **f** Representative pictures of day 8 uteri (D) and implantation site (IS)-positive rate (**e**) for each genotype on day 8 of pregnancy. Unattached blastocysts (right) were confirmed by uterine flushing in *Lif* eKO and uKO mice. Percentages of IS-positive rates and numbers of females with implantation sites/total tested are noted above the bars in (**g**).

Lif is expressed in the uterine glands before embryo attachment, and on day 5 after embryo attachment, it is induced in the stroma surrounding the attached embryos (Fig. 1a). To investigate the detailed mechanisms by which glandular and stromal Lif contribute to early pregnancy events, we established *Lif* eKO and *Lif* uKO mice by mating *Lif-loxP* with *Ltf-iCre* or *Pgr-Cre* drivers, respectively (Fig. 1b). *Lif* mRNA was efficiently deleted in target cells by both Cre drivers (Fig. 1c, d). We first determined the pregnancy rates in the mutant females. While seven out of nine females produced pups in the control group (7.0 ± 1.2 pups/litter, mean ± SD), only one *Lif* eKO female gave birth (six pups) and the remaining six females did not produce offspring. Furthermore, *Lif* uKO females completely failed to have pups (Fig. 1e).

These severe infertility phenotypes in the *Lif* mutants led us to observe the early pregnancy events in these milieus. We sacrificed females on day 8 of pregnancy when embryo implantation sites become evident [18]. While implantation sites appeared in 100% of control pregnant females, they were only evident in 25% of *Lif* eKO uteri; furthermore, these implantation sites were smaller than those in control uteri, whereas 75% of *Lif* eKO and 100% of *Lif* uKO uteri never developed implantation sites (Fig. 1f, g). Notably, the implantation-failed uteri contained blastocysts packed with inner cell masses and enlarged trophectoderm, which are characteristic features of diapaused blastocysts [22]. These results suggest that early pregnancy events, particularly embryo activation and attachment, are compromised by *Lif* deletion in the uterus.

### *Lif* uKO mice do not defect endometrial receptivity but embryo attachment

**Fig. 2:**
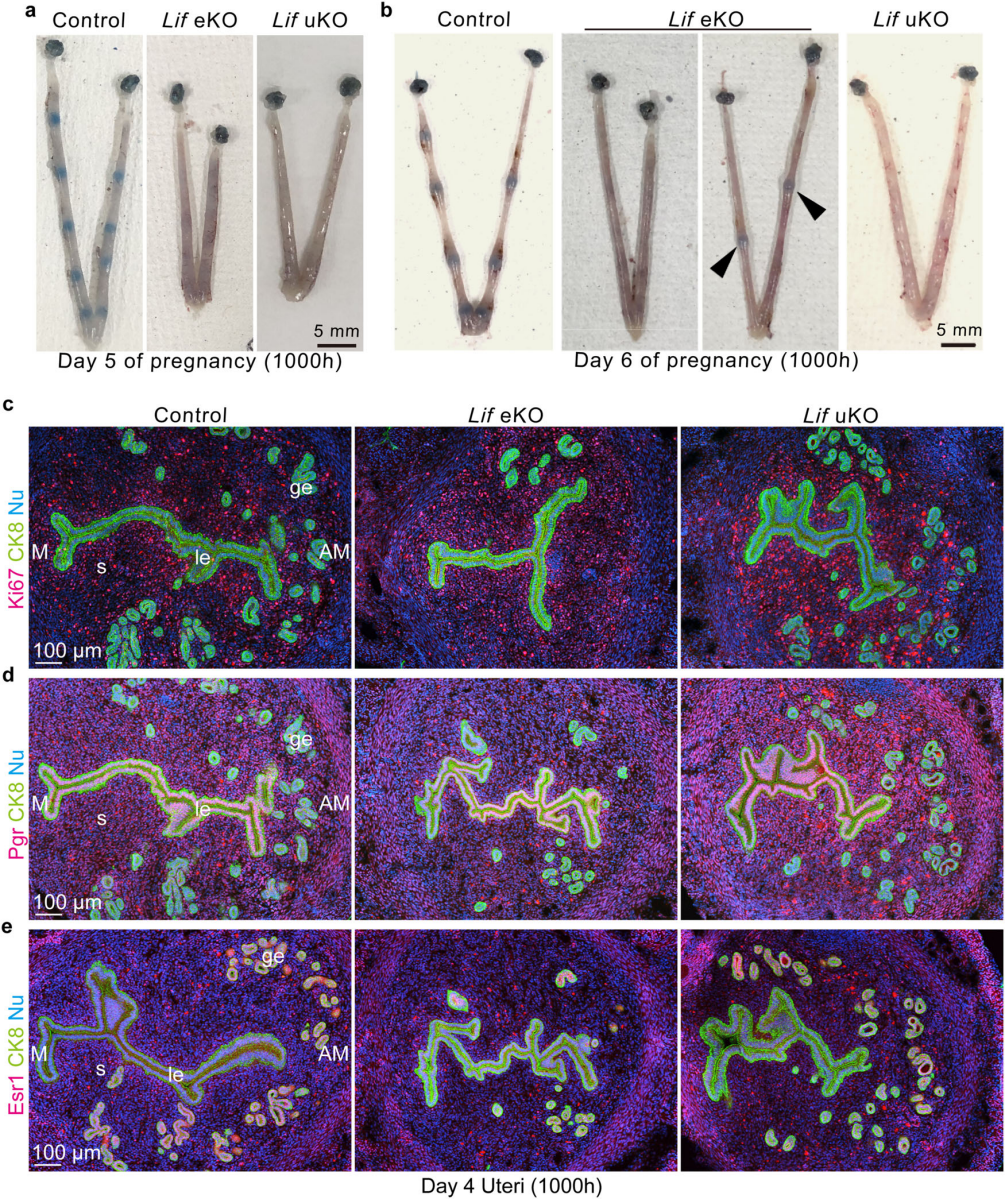
Embryo attachment, but not uterine receptivity, is impaired upon Lif deletion. **a, b** Representative images of pregnant uteri of each genotype on day 5 (**a**) and day 6 (**b**). Arrowheads indicate faint implantation sites. Scale bar: 5 mm. *n* = 4 for each image. **c**–**e** Immunostaining of Ki67 (a cell proliferation marker; **c**) Pgr (P_4_ receptor; **d**), and Esr1 (E_2_ receptor; **e**) indicated that uterine receptivity was not affected by *Lif* deletion in the uterus. CK-8 (an epithelial marker) was stained to visualize epithelial cells. M mesometrium, AM anti-mesometrium, le luminal epithelium, ge glandular epithelium, s stroma. Scale bar: 100 µm. *n* = 3 for each image.

Considering the unattached blastocysts in *Lif-*deficient uteri on day 8, we traced implantation events back to earlier pregnancy days. Normally, the endometrium surrounding attached embryos undergoes decidualization, which is accompanied by increased vascular permeability [23]; hence, implantation sites can be observed by injecting a blue dye on day 5 of pregnancy (Fig. 2a, b). Most *Lif* eKO uteri revealed the lack of implantation sites on day 5 and the morning of day 6 (Fig. 2a, b). Furthermore, implantation sites were not observed in *Lif* uKO uteri (Fig. 2a, b), confirming that *Lif* is critical for embryo attachment and subsequent implantation processes.

We and other researchers have previously demonstrated that proliferation-differentiation switching (PDS) in endometrial cells is crucial for acquiring receptivity against implantation-competent blastocysts [4]. Stromal cells proliferate from day 3 onwards in response to increasing serum progesterone (P_4_) levels, whereas estrogen (E_2_)-induced epithelial growth ceases. Sustained epithelial proliferation often accompanies poor stromal growth in the peri-implantation period, resulting in failed embryo implantation [24–26]. However, we observed comparable levels of stromal proliferation between *Lif*-deficient and control uteri, with poor epithelial growth on day 4, as evidenced by Ki67 immunostaining (Fig. 2c). In line with the normal PDS, the localization of receptors for P_4_ (Pgr; Fig. 2d) and E_2_ (Esr1; Fig. 2e) was not impaired by *Lif* uKO mice. Additionally, serum P_4_ levels were comparable on days 4 and 8, indicating normal ovarian function in the mutants (Supplementary Fig. 1). These data suggest that Lif contributes to embryo attachment without affecting endometrial receptivity.

### Lif-stimulated transition of the uterine epithelium from receptive to embryo-attaching phases

**Fig. 3:**
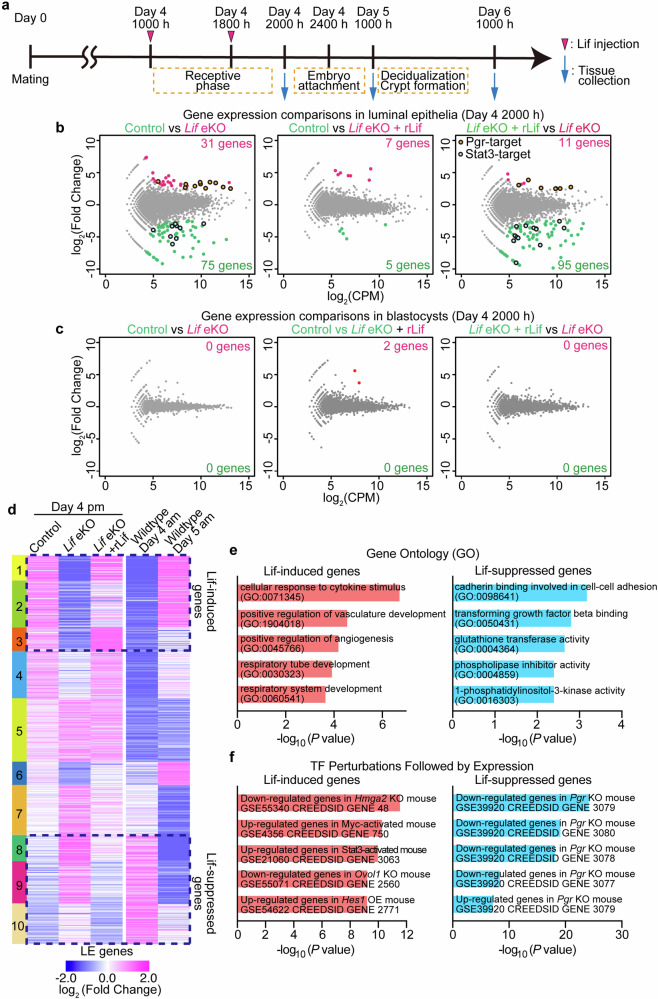
Epithelial gene expression regulated by Lif during the receptive phase. **a** Schematic diagram of the experiments of which the data are presented in Figs. 3–5. Uterine tissues were collected on day 4 evening (Fig. 3), day 5 morning (Fig. 4), and day 6 morning (Fig. 5), respectively. For rescue experiments, mutant females received rLif during the receptive phase, when the uterine glands produce Lif in wild-type mice. **b**, **c** MA plots depicting DEGs in luminal epithelia (**b**) and blastocysts (**c**) on day 4 evening. Control (floxed females), *Lif* eKO, and rLif-treated *Lif* eKO mice are compared in each plot. Significantly upregulated genes and their numbers in each experimental group are highlighted in magenta or green. Among the DEGs, genes targeted by Pgr- or Stat3 are encircled by black lines. **d** Heatmap depicting DEGs with a > 2-fold change and a false discovery rate < 0.05 identified using edgeR. Data from luminal epithelia on day 4 evening from each group were compared with that of luminal epithelia on day 4 and day 5 morning, which we previously reported [27]. A total of 697 DEGs were classified into 10 clusters using k-means clustering. **e** Top 5 GO terms determined using Enrichr for Lif-induced genes on day 5 (clusters 1 and 2) and Lif-suppressed genes on day 4 (clusters 3 and 4). **f** Upstream transcription factors for the clusters were predicted by enrichment analysis in comparison with “TF_Perturbations_Followed_by_Expression” using Enrichr.

We investigated the mechanisms by which uterine Lif promotes endometrial interaction with blastocysts. To this end, we performed RNA-sequencing (RNA-seq) analysis of luminal epithelia and blastocysts collected from *Lif* eKO uteri on day 4 evening, right before embryo attachment. To identify genes affected by the presence of Lif during the receptive phase, we treated *Lif* eKO mice with rLif (Fig. 3a). We identified differentially expressed genes (DEGs) between control and *Lif* eKO epithelia (Fig. 3b and Tables 1–3). The *Lif* eKO-induced expression changes were largely reversed upon rLif treatment (Fig. 3b and Tables 1–3). Notably, expression changes were negligible in exogenous Lif-treated blastocysts (Fig. 3c), implying that Lif does not directly stimulate blastocysts to attach to the endometrium.

We monitored the DEGs within the epithelium influenced by Lif. We recently reported dynamic gene expression changes in the luminal epithelium before and after embryo attachment [27]. To validate our findings, we compared the current dataset with changes in epithelial gene expression pre- and post-embryo attachment on days 4 (morning) and 5 (morning). Consequently, distinct expression patterns emerged based on the presence of Lif (Fig. 3d and Table 4): The *Lif* eKO epithelium showed similar expression patterns on day 4 morning, whereas rLif treatment counteracted this effect. Genes in clusters 1 − 3, highly expressed in the presence of Lif, were designated as Lif-induced genes. Conversely, clusters 8 − 10, corresponding to genes expressed in the *Lif*-depleted epithelium on day 4 evening, were termed Lif-suppressed genes. These clusters were also enriched on the day 4 morning epithelium, but not on the day 5 morning epithelium. Enrichment analysis using Enrichr helped identify unique Gene Ontology (GO) terms in each gene group (Fig. 3e, f and Table 5). Notably, the identified GO terms in each group were similar to those of DEGs in the luminal epithelium between receptive and embryo attachment phases, as previously reported [27] (Fig. 3e). Specifically, Lif-induced genes were enriched in GO terms related to cytokine signaling and angiogenesis (Fig. 3e, left panel), similar to findings for the luminal epithelium post-attachment [27]. Conversely, Lif-suppressed genes on day 4 morning were enriched in GO terms related to cell−cell adhesion and glutathione metabolism (Fig. 3e, right panel), which were also enriched in the epithelium during days 3 and 4, before attachment [27].

Further enrichment analysis helped identify transcriptional factors regulating gene expression downstream of Lif (Fig. 3f). Comparison with the published gene dataset in GEO using Enrichr indicated Stat3 association with Lif-induced genes, in alignment with its role as a critical downstream factor of Lif in the uterus [12, 28] (Fig. 3b f, left panel). Notably, Lif-suppressed genes highly correlated with genes downregulated in *Pgr*-deleted mice (Fig. 3b, f, right panel), suggesting that Lif suppresses Pgr-induced genes. This result resonates with the notion that sustained or excessive P_4_-Pgr signaling disturbs embryo attachment [19, 29], corroborating the hypothesis of Lif facilitating blastocyst attachment to the endometrium. Collectively, our findings suggest that Lif orchestrates epithelial transition from receptivity to the attachment phase, without exerting a direct effect on blastocysts.

### Epithelial Lif–Stat3 axis is indispensable for blastocyst attachment

**Fig. 4:**
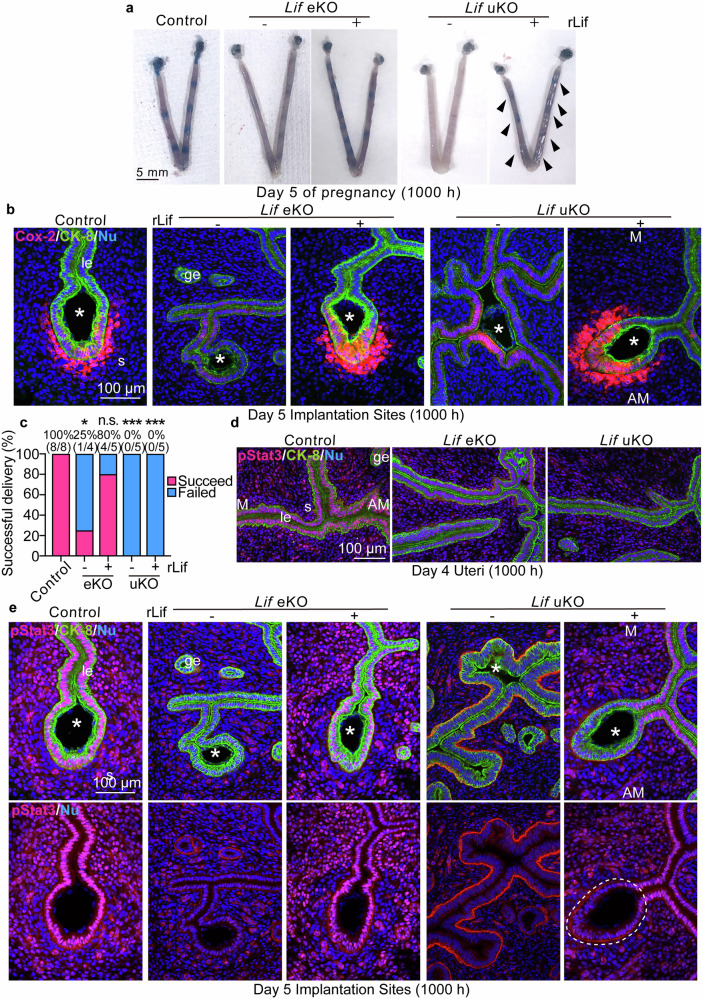
Lif expressed during the receptive phase facilitates embryo attachment but is not sufficient for complete activation of Stat3 in the epithelial crypts. **a** Representative images of day 5 uteri from each genotype. *Lif* eKO and uKO females were treated with rLif as described in Fig. 3a. Arrowheads indicate faint blue staining. Scale bar: 5 mm. *n* = 4 for each image. **b** Immunostaining of Cox-2 revealed improved embryo attachment in *Lif-*deficient uteri treated with rLif. CK-8 was co-stained as an epithelial marker. Asterisks indicate blastocysts. le: luminal epithelium, ge: glandular epithelium, s: stroma, M: mesometrial pole, AM: anti-mesometrial pole. Scale bar: 100 µm. *n* = 4 for each image. **c** Percentages of pregnancy rates and numbers of female mice with successful pregnancy/total tested are presented above the bars. *P < 0.05, ***P < 0.001, n.s.: not significant by Fisher’s exact test comparing each group with control. **d**, **e** Immunostaining of pStat3 and CK-8 on day 4 morning (**d**) and day 5 morning (**e**). The dashed line indicates poor pStat3 localization in the crypt epithelia in *Lif* uKO uteri, even after rLif treatment. Asterisks indicate blastocysts. le luminal epithelium, ge glandular epithelium, s stroma, M mesometrial pole, AM anti-mesometrial pole. Scale bar: 100 µm. *n* = 4 for each image.

We further investigated the functional roles of Lif in the endometrium. As outlined in Fig. 3a, we treated *Lif* mutant mice with rLif during the receptive phase and collected uteri on day 5 morning. Exogenous Lif increased blue staining intensity in *Lif* eKO and uKO uteri (Fig. 4a), although the staining was faint in *Lif* uKO uteri. Cyclooxygenase-2 (Cox-2) is induced in the luminal epithelium and stroma in the vicinity of attached embryos [2, 30]; therefore, it is often used as a marker of embryo attachment. Compared with the distinct Cox-2 signal in control uteri, *Lif* eKO and uKO uteri revealed only weak expression of this protein in the luminal epithelium (Fig. 4b). This aberrant Cox-2 expression was rescued by rLif injection in the mutant mice (Fig. 4b), indicating that Lif supplied during the receptive phase promotes embryo attachment, which corroborates with the RNA-seq data (Fig. 3). However, we found that rLif could recover delivery rates only in *Lif* eKO mice, but not in *Lif* uKO mice (Fig. 4c), indicating that the stromal Lif is crucial for the pregnancy maintenance after embryo attachment.

Supporting this notion, we observed that Stat3 activation required both epithelial and stromal induction of Lif, while epithelial Lif alone was sufficient to evoke attachment (Fig. 4d, e). Stat3 is a transcription factor activated downstream of tyrosine-kinase receptors and Janus kinases [7]. Phosphorylated (p)Stat3 translocates to the nucleus to exert its transcriptional activity. Considering the RNA-seq data indicating dysregulation of Stat3 signaling under Lif deficiency (Fig. 3f), we verified Stat3 activation in the mice (Fig. 4c, d). As previously observed, control uteri exhibited distinct localization of pStat3 in the luminal epithelium on day 4 morning (Fig. 4c, left panel), whereas pStat3 was poorly detected in *Lif* eKO and uKO uteri (Fig. 4c, middle and right panels), suggesting that Lif activates Stat3 signaling during the receptive phase.

We then examined pStat3 in the day 5 endometrium after rLif injection. In control uteri, pStat3 was distinctly localized in nuclei in the luminal epithelium and stroma surrounding attached blastocysts (Fig. 4d). Similar to day 4 uteri, *Lif*-deleted uteri exhibited defective Stat3 activation on day 5. However, rLif treatment during the receptive phase recovered Stat3 phosphorylation in *Lif* eKO uteri. Notably, in *Lif* uKO uteri, rLif rescued Stat3 activation, except in the embryo-surrounding epithelium. Considering that stromal Lif is induced in the vicinity of embryos after attachment [9], the absence of stromal Lif may have caused this defective Stat3 activation in the *Lif* uKO uterus.

Collectively, these findings revealed that epithelial Lif facilitates embryo attachment during the receptive phase but is not sufficient to induce Stat3 activation in the embryo-attached epithelium.

### Epithelial Lif–Stat3 axis primes implantation chamber formation, which is dependent on the presence of blastocysts

**Fig. 5:**
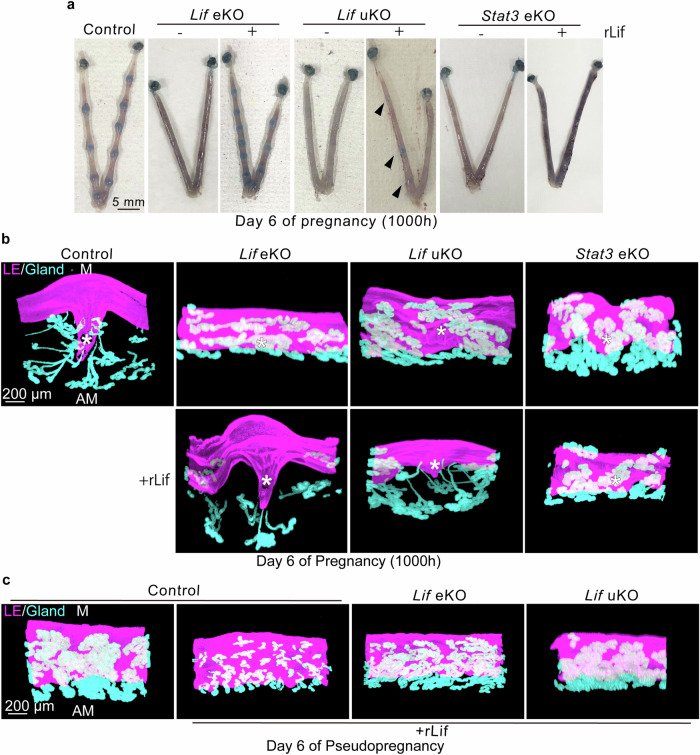
Lif-induced embryo attachment further promotes epithelial crypt formation, which is dependent on stromal Lif and epithelial Stat3. **a** Representative images of day 6 uteri from each genotype. *Lif* eKO and uKO females were treated with rLif during the receptive phase, as outlined in Fig. 3a. Arrowheads indicate faint blue staining. Scale bar: 5 mm. *n* = 4 for each image. **b** 3D visualization of E-cadherin-stained epithelia indicating that implantation chamber (crypt) formation is dependent on the Lif–Stat3 axis. Asterisks indicate the positions of blastocysts. M: mesometrial pole, AM: anti-mesometrial pole. Scale bar: 200 µm. *n* = 3 for each image. **c** 3D visualization of epithelia in pseudopregnant uteri demonstrates that Lif-induced crypt formation requires the presence of blastocysts. M mesometrial pole, AM anti-mesometrial pole. Scale bar: 200 µm. *n* = 3 in each image.

We next investigated the mechanisms by which aberrant Stat3 activation contributes to the subsequent pregnancy phase. On day 6 of pregnancy, we observed blue-dye reactions in *Lif*-deleted females treated with rLif during the receptive phase (Figs. 3a and 5a). Similar to uteri on day 5, *Lif* eKO uteri failed to exhibit blue staining, which was efficiently recovered using rLif treatment (Fig. 5a). In contrast, rLif produced only faint staining in *Lif* uKO uteri (Fig. 5a). We observed failed embryo implantation in *Stat3* eKO mice (*Stat3*^*f/f*^*Ltf*^*Cre/+*^) (Fig. 5a), as previously reported [15]. rLif failed to produce blue staining reactions in *Stat3* eKO mice (Fig. 5a), supporting our hypothesis that Lif contributes to embryo implantation via the activation of epithelial Stat3.

Subsequently, we investigated whether the Lif–Stat3 axis affects the epithelial morphology, which is critical for implantation success [5, 6]. Whole-mount tissue staining allows 3D visualization of the uterine epithelium [5, 6]. After embryo attachment, the luminal epithelium surrounding blastocysts creates deep invaginations into the anti-mesometrial pole, with well-extended glands. Accumulating studies using 3D visualization have demonstrated that abnormal crypt formation can impair subsequent pregnancy processes [6, 16, 26, 31]; however, the gene networks governing epithelial morphology remain unclear. We investigated 3D views of the uterine epithelium on day 6 morning, when clear crypts and extended glands should be observed around attached blastocysts (Fig. 5b) [6]. In contrast to the well-developed crypts in control uteri, *Lif* eKO and uKO uteri failed to undergo epithelial morphological transformation (Fig. 5b, upper panel). Notably, while blastocysts collocated on the anti-mesometrial side in *Lif* eKO uteri, the luminal epithelium in *Lif* uKO uteri appeared unclosed, with blastocysts floating in the center of the luminal cavity (Fig. 5b, upper panel and Supplementary Movies 1–4). These different phenotypes between eKO and uKO mice may have been caused by the remaining stromal Lif in *Lif* eKO mice. Similarly, *Stat3* eKO mice abrogated the transformation of the epithelium surrounding blastocysts, with cylindrical luminal layers and curled glands (Fig. 5b, upper panel). rLif treatment during the receptive phase ameliorated crypt formation in *Lif* eKO mice on day 6 (Fig. 5b, lower panel, Supplementary Movies 5). However, *Lif* uKO mice exhibited poor chamber formation even after rLif treatment, although the embryo was located on the anti-mesometrial pole (Fig. 5b, lower panel, Supplementary Movies 6). These differential effects of exogenous Lif in the different Lif mutants demonstrated that epithelial Lif evokes embryo attachment but is insufficient for full implantation chamber formation in the absence of stromal Lif surrounding the attached blastocysts. Furthermore, we did not observe crypt formation in *Stat3* eKO mice, even after rLif injection (Fig. 5b, lower panel, Supplementary Movies 7), indicating that Lif requires subsequent Stat3 activation in the epithelium to exert its functions during the peri-implantation period. Notably, rLif treatment in pseudopregnant females did not induce crypt formation (Fig. 5c). These observations suggest that the Lif–Stat3 axis triggers the epithelium to form embryo implantation chambers in response to the presence of blastocysts.

## Discussion

**Fig. 6:**
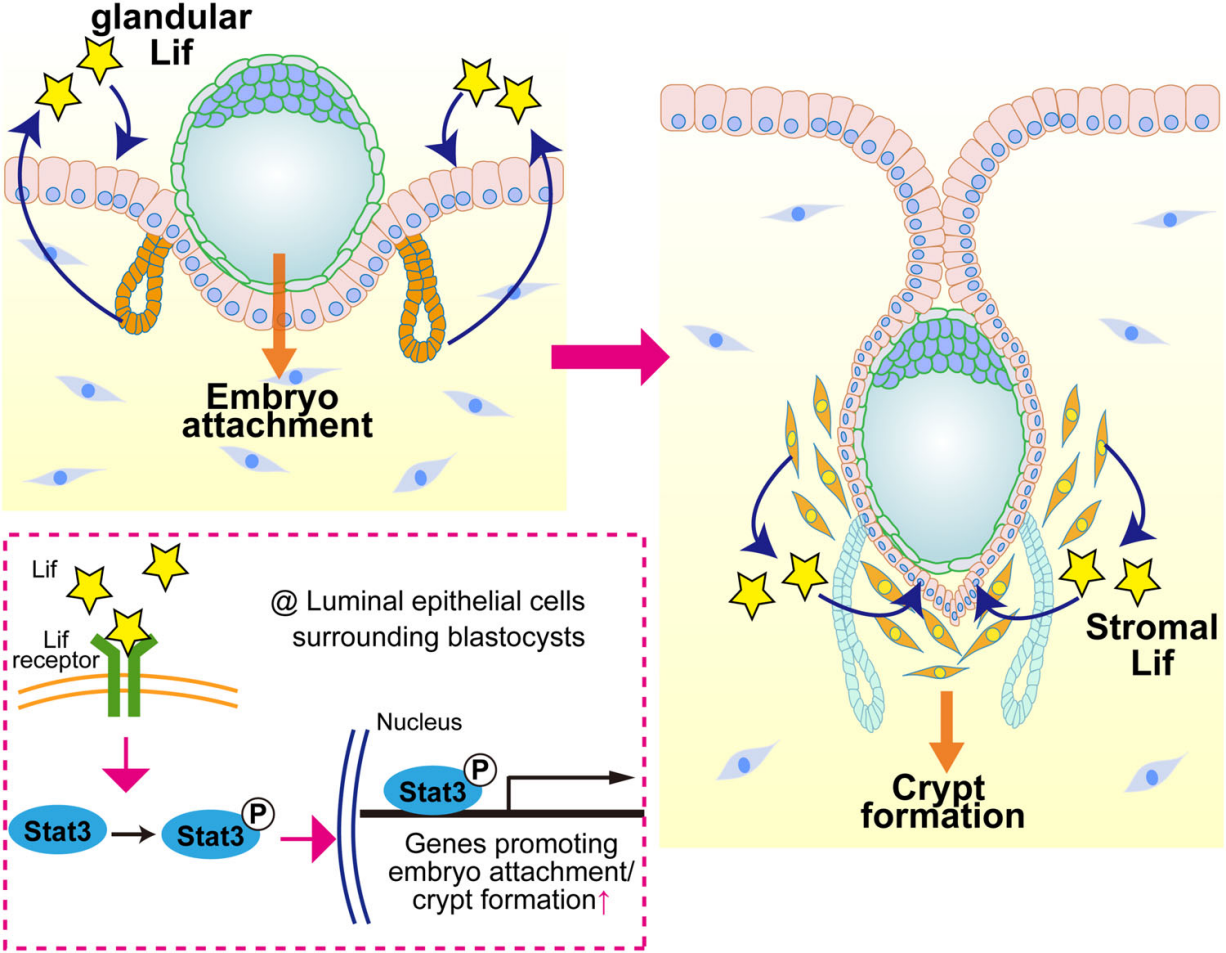
Schematic diagram of the spatiotemporal functions of Lif in the uterus during the peri-implantation period. During the receptive phase (day 4), glandular Lif activates Stat3 in the luminal epithelium, facilitating gene expression required for embryo attachment. Once the embryo is attached, stromal Lif is induced in the vicinity of blastocysts to promote crypt formation by activating Stat3 in the embryo-attached epithelium.

In this study, we elucidated the uterine physiological role of Lif using mice with conditional KO of *Lif* in the uterine epithelium or the whole uterus. *Lif* eKO mice compromised the transition of epithelial gene expression from the receptive to the embryo attachment phase, resulting in aberrant crypt formation. *Lif* deletion in both the epithelium and stroma further abrogated early pregnancy events, with failed luminal closure. We previously reported that mice with eKO of *Lifr* and *Stat3* resulted in infertile phenotypes with abnormal epithelial morphology [15, 16]. In this study, we observed normal PDS in *Lif* eKO and uKO mice, indicating that the anomalies in the *Lif-*deleted epithelium are not due to sustained proliferation of epithelial cells. Similarly, *Lifr* eKO/uKO and *Stat3* eKO uteri also maintained normal PDS [15, 16], whereas mice lacking uterine *Gp130* or stromal *Stat3* have defective PDS in the receptive phase [12, 15]. These data suggest that Lif regulates epithelial morphology via the epithelial Lifr–Stat3 axis (Fig. 6). Stromal Gp130 and Stat3 may be activated by other cytokines to contribute to appropriate PDS during the blastocyst-receptive phase.

Experiments using exogenous Lif revealed the differential roles of Lif before and after embryo attachment. Glandular epithelial cells produce high levels of Lif during the receptive phase. Lif expression is then induced in the stroma surrounding attached embryos [9]. In *Lif* eKO mice, exogenous Lif treatment during the receptive phase ameliorated embryo-chamber formation, whereas in *Lif* uKO mice, it only improved embryo attachment. These results demonstrate that a first wave of epithelial Lif is critical for embryo attachment but insufficient to cause crypt formation in the absence of stromal Lif. Exogenous Lif injections could not produce epithelial crypts in pseudopregnant uteri; hence, the presence of blastocysts is critical to prime the second wave of Lif. Intriguingly, RNA-seq analysis indicated that Lif does not directly trigger blastocysts to implant, whereas Lif-induced chamber formation requires blastocysts. As Lif activates Stat3 signaling in the luminal epithelium upon embryo attachment, Stat3 may be key to inducing certain secretory molecules that stimulate blastocysts. In agreement with our hypothesis, pStat3 levels were suppressed in the Lif-deficient epithelium. In addition, Lif-deficient uteri and *Stat3* eKO uteri exhibited similar anomalies in crypt formation, with poor evagination of the luminal epithelium. While Lif is dispensable for gene expression regulation upon embryo attachment, blastocysts underwent diapause the absence of uterine Lif (Fig. 1f). Lif supplementation in delayed implantation reportedly activates dormant blastocysts, causing embryo implantation [22]. Hence, Lif likely influences gene expression in blastocysts once they undergo diapause. Investigating uterine and embryonic transcriptomes during delayed and activated implantation in Lif–Stat3-deleted uteri would yield valuable insights.

While our current findings unveiled previously unknown mechanisms of uterine Lif using *Lif* eKO and uKO mice, it remains unclear how stromal Lif functions following embryo attachment, particularly due to the lack of uterine stromal-specific deletion of Lif. In contrast to *Lif* uKO mice, which are completely infertile, some *Lif* eKO mice can progress through all stages of pregnancy to delivery (Figs. 1e and 4c). This may be attributed to the upregulation of stromal Lif in this context (Fig. 1d), although the increase in mRNA levels in *Lif* eKO mice is not statistically significant. Additionally, we found rLif treatment during the receptive phase can recover delivery rates in *Lif* eKO mice, but not *Lif* uKO mice, suggesting that stromal Lif plays a role in maintaining healthy pregnancy outcomes, including decidualization. It is also uncertain whether this system is conserved in other species, including humans. Uterine LIF has been identified in mammals beyond mice [21, 32, 33]. Human studies indicate LIF expression in uterine glands, peaking during the mid-secretory phase [21, 32] when the endometrium is receptive to blastocysts. However, our study had the following limitation: ethical considerations impede direct analysis of implantation sites in humans, the role of stromal LIF in the human uterus remains unclear. Distinct modes of embryo attachment and invasion in rodents and primates highlight the potential importance of glandular LIF in the human uterus. Histological research by Enders et al. demonstrated direct contacts between invading blastocysts and glands during embryo attachment to decidualization stages (https://www.trophoblast.cam.ac.uk/Resources/enders). The expressional and functional traits of uterine LIF across species warrants further investigation.

In conclusion, we demonstrated the spatiotemporal roles of uterine Lif in ensuring embryo attachment and crypt formation in the mouse uterus. Epithelial Lif is pivotal for facilitating embryo attachment, followed by the formation of deeply crypted epithelial chambers driven by blastocysts, potentially through stromal Lif induction. In the epithelium, Lif activates Stat3 via phosphorylation, promoting embryo implantation. Nevertheless, the mechanism by which the Lif-Stat3 axis triggers blastocyst activation from diapause warrants further investigation.

## Methods

### Generation of *Lif*-loxP mice

A *Lif* targeting vector (*Lif*^*tm1e(EUCOMM)Wtsi*^ vector) was provided by the European Conditional Mouse Mutagenesis Program (EUCOMM). In the targeting vector, *Lif* exon 3 and part of exon 4 are flanked with loxP sites, and an L1L2_Bact _P cassette flanked by flippase recognition target (FRT) sites is inserted between exons 2 and 3 (Supplementary Fig. 2). EGF-G01 ES cells were electroporated with the targeting vector and selected using G418 and ganciclovir (Thermo Fisher Scientific, Waltham, MA, USA), as reported previously [34]. These ES cells were used to generate chimeras through injection into blastocysts from C57BL/6 N females (SLC, Shizuoka, Japan). To obtain chimeric mice, these blastocysts were transferred into pseudopregnant ICR wild-type females (SLC). The chimeric mice were mated with wild-type C57BL/6 N mice (SLC). F1 agouti offspring were analyzed for homologous recombination and crossed with CAG-Flpo mice to remove the L1L2_Bact _P cassette flanked by FRT sites and establish *Lif-loxP*/+ mice [35] (Supplementary Fig. 2). *Lif-loxP*/+ mice were intercrossed to generate *Lif-loxP/loxP* mice containing homozygous recombinant alleles.

### Generation of *Lif* uKO and eKO mice

*Lif-loxP/loxP*, *Ltf-iCre*, and *Pgr-Cre* mice were used in this study. Lactoferrin (Ltf) is expressed in the uterine epithelium [36], whereas progesterone receptor (Pgr) is expressed in the entire uterus (i.e., epithelium, stroma, and myometrium) [37] (Fig. 1b). *Lif-loxP/loxP* females were crossed with *Ltf-iCre* and *Pgr-Cre* males to generate mice with deletion of *Lif* in the epithelium (*Lif* eKO mice) or the whole uterus (*Lif* uKO mice), respectively.

### Evaluation of pregnancy outcomes

To examine pregnancy outcomes, *Lif* eKO, *Lif* uKO, or *Lif-loxP/loxP* female mice were mated with C57BL/6 N fertile male mice, as previously reported [15, 16]. The day when the vaginal plug was detected was considered day 1 of pregnancy. Pregnant mice were euthanized by cervical dislocation on designated days of pregnancy for the evaluation of pregnancy phenotypes and sample collection. On day 4, one uterine horn was flushed with saline to confirm the presence of blastocysts. Embryo attachment sites were observed as blue bands promptly after intravenous injection of 1% solution of Chicago blue dye (Sigma Aldrich, St Louis, MO, USA) in saline as of day 5 [23]. When no embryo attachment sites were observed, uterine horns were cut and flushed with saline to collect embryos. All mice were housed in the University of Tokyo Animal Care Facility according to the institutional guidelines for the use of laboratory animals.

### rLif treatment

rLif injections were performed as previously reported [24]. The rLif expression vector was kindly gifted by Prof. Eichi Hondo [38]. To determine the function of Lif, female mice received rLif (20 µg/head, *i.p*.) at 10 am and 6 pm of day 4 of pregnancy or pseudopregnancy. For RNA-seq analyses, mice were dissected 2 h after the last injection. Otherwise, tissues were collected on day 5 or 6 morning as outlined in Fig. 3a.

### Isolation of mouse uterine epithelial and stromal cells

Uterine epithelium and stroma were collected as previously reported [26, 31]. Briefly, on day 4 morning, mouse uteri were digested in 25 mg/mL pancreatin (Sigma Aldrich) and 6 mg/mL dispase in DMEM/F12 (Gibco, Waltham, MA, USA). The tissues were incubated at 4 °C for 1 h, room temperature for 1 h, and at 37 °C for 10 min. Epithelial cells were collected and incubated in TRI Reagent (Molecular Research Center, Cincinnati, OH, USA) for RNA extraction. The remaining tissues were further digested in 10 mg/mL collagenase (Fujifilm Wako, Osaka, Japan). After filtration through a 70-µm mesh (Falcon, Corning, NY, USA), the cells were cultured in DMEM/F12 containing 10% charcoal-stripped fetal bovine serum (Hyclone, Logan, UT, USA) in a 60-mm dish (Thermo Fisher scientific). After 30 min, the medium was refreshed to remove immune cells. After an additional culture for 5.5 h, stromal cells attached to the dish were suspended in TRI Reagent.

### RNA extraction and quantitative reverse transcription

RNA was extracted from homogenized tissues using TRI Reagent (Molecular Research Center) according to the manufacturer’s protocol. The quality and quantity of extracted RNA were examined by Nanodrop (Thermo Fisher Scientific). cDNA was synthesized from the extracted RNA using ReverTra Ace qPCR RT Master Mix with gDNA Remover (TOYOBO, Shiga, Japan). qPCR was performed using the THUNDERBIRD SYBR qPCR Mix (TOYOBO). The housekeeping gene *Actb* was used for internal standard normalization. Relative expression levels were determined using the ΔΔCt method [39]. The primer sequences were as follows: *Lif* 5′-GCTATGTGCGCCTAACATGA-3′ and 5′-AGTGGGGTTCAGGACCTTCT-3′; *Actb* 5′-TGTTACCAACTGGGACGACA-3′ and 5′-GGGGTGTTGAAGGTCTCAAA-3′.

### Embryo collection for RNA-seq

Females of each genotype were sacrificed on day 4 evening as outlined in Fig. 3a. Blastocysts were collected from the uterine horns by flushing with PBS. Forty-four blastocysts from eight control mice, 46 from *Lif* eKO mice, and 51 from *Lif* eKO mice treated with rLif were collected. The blastocysts were pooled into one sample per experimental group. RNA was extracted using a NucleoSpin RNA kit (Takara, Osaka, Japan) according to the manufacturer’s protocol.

### Laser microdissection of the epithelium

Uterine tissues were collected from *Lif-*floxed, *Lif* eKO, and rLif-treated *Lif* eKO mice at 8 pm on day 4. One side of the uterine horn was flushed with PBS to collect blastocysts and confirm pregnancy. The other side of the uterine horn was snap-frozen for cryosectioning. Laser microdissection was performed as described previously [18, 27]. Luminal epithelia of each section were microdissected using an LMD7000 system (Leica Microsystems, Wetzlar, Germany). For each group, RNAs from three independent samples were pooled into one sample for RNA-seq.

### RNA-seq

RNA extracted from laser microdissection samples or blastocysts was processed using a SMART-seq v.4 Ultra Low Input RNA Kit (Takara) and subjected to RNA-seq using the BGI RNA-seq service (BGI, Hong Kong, China; www.bgi.com) according to the standard protocol. The data were analyzed as previously reported [27]. In brief, raw paired-end RNA-seq reads were aligned to indexed mouse genome (GRCm38/mm10) by HiSAT2 [40]. The reads per kilobase of exon per million mapped sequence reads were counted using the FeatureCounts function in Subread (v.2.0.0) [41]. MA plots were generated and k-means clustering performed using trinityrnaseq (v.2.0.6) [42]. DEGs were defined based on a log2 fold change > |1| and false discovery rate < 0.05 using edgeR. Heatmaps of the DEGs were created using Morpheus (https://software.broadinstitute.org/morpheus/). Genes in each cluster were subjected to comparative and GO analyses using Enrichr (https://amp.pharm.mssm.edu/Enrichr/) [43].

### Immunofluorescence

Frozen sections (12 μm) were used for immunofluorescence. After fixation in 4% paraformaldehyde in PBS, the sections were incubated with primary antibodies to Ki67 (Thermo Fisher Scientific, SP6, 1/300), Pgr (8757, 1:300; Cell Signaling Technology, Danvers, MA, USA), Esr1 (ab32063, 1:300; Abcam, Cambridge, UK), Cox-2 (160106, 1:300; Cayman, Ann Arbor, MI, USA), pStat3 (ab76315, 1:100; Abcam), and CK8 (TROMA-I, 1:300; DSHB, Iowa city, IA, USA). Signals were detected using Alexa Fluor 555-conjugated anti-rabbit immunoglobulin G (A21428, 1:500; Thermo Fisher Scientific), Alexa Fluor 488-conjugated anti-rat immunoglobulin G (A11006, 1:500; Thermo Fisher Scientific), and 4, 6-diamidino-2-phenylindole (1:500; Dojindo, Kumamoto, Japan). Images were acquired using an AXR confocal microscopy system (Nikon, Tokyo, Japan). Quantification of immunostaining of the target protein per each cell type (Supplementary Fig. 3) was performed using ImageJ (NIH).

### 3D visualization of implantation sites

3D visualization of day 6 implantation sites was performed as previously reported [6]. To stain luminal and glandular epithelial cells, day 6 tissues were incubated with an anti-E-cadherin antibody (24E10, 1:500; Cell Signaling Technology) and then with an anti-rabbit antibody conjugated with Alexa 555 (A21428, 1:500; Thermo Fisher Scientific). 3D images were acquired using LSM 880 (Carl Zeiss, Oberkochen, Germany) and AXR (Nikon) microscopes. To construct a 3D structure from the images, the surface tool in Imaris (v.9.8, Oxford instruments, Abingdon-on-Thames, UK) was used.

### Measurement of serum P_4_ levels

Blood samples were collected from mice on the indicated days of pregnancy. Serum P_4_ levels were measured as described previously [18], using a Progesterone EIA kit (582601, Cayman).

### Statistical analysis

Statistical analyses were performed using a two-tailed Student’s *t*-test or one-way ANOVA followed by Bonferroni post-hoc tests in Prism9 (GraphPad, San Diego, CA, USA). Significance was set at *P* < 0.05.

## Supplementary information

Supplementary_Information

Supplementary Movie 1

Supplementary Movie 2

Supplementary Movie 3

Supplementary Movie 4

Supplementary Movie 5

Supplementary Movie 6

Supplementary Movie 7

Table 1

Table 2

Table 3

Table 4

Table 5

## Data Availability

RNA-seq data reported in this study were deposited to the Gene Expression Omnibus (accession no. GSE 253371 and GSE254815).
