# Supplementary material for: Spatiotemporal functions of leukemia inhibitory factor in embryo attachment and implantation chamber formation
Source: Cell Death Discov. 2024 Nov 25;10:481. doi: 10.1038/s41420-024-02228-4 (PMC11589870; doi:10.1038/s41420-024-02228-4)
Supplement: Supplementary file 1 — Supplementary_Information [file 41420_2024_2228_MOESM1_ESM.docx]

# Supplementary figures

##
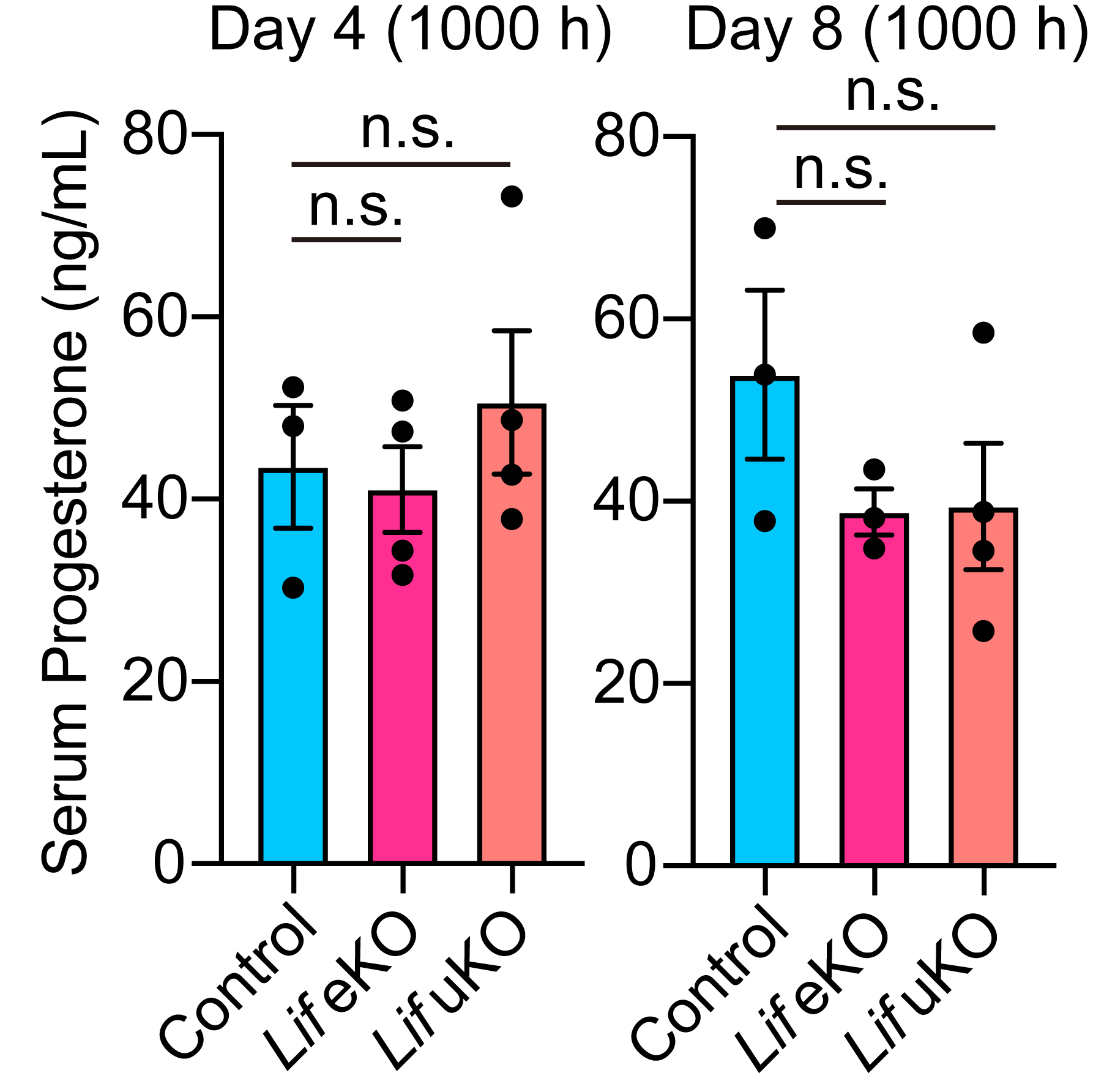


## Supplementary Figure 1. Comparable serum P_4_ levels in each genotype.

Sera were collected in the morning on day 4 or 8 of pregnancy. n = 3−4 for each genotype. n.s.: not significant (Student’s *t*-test).


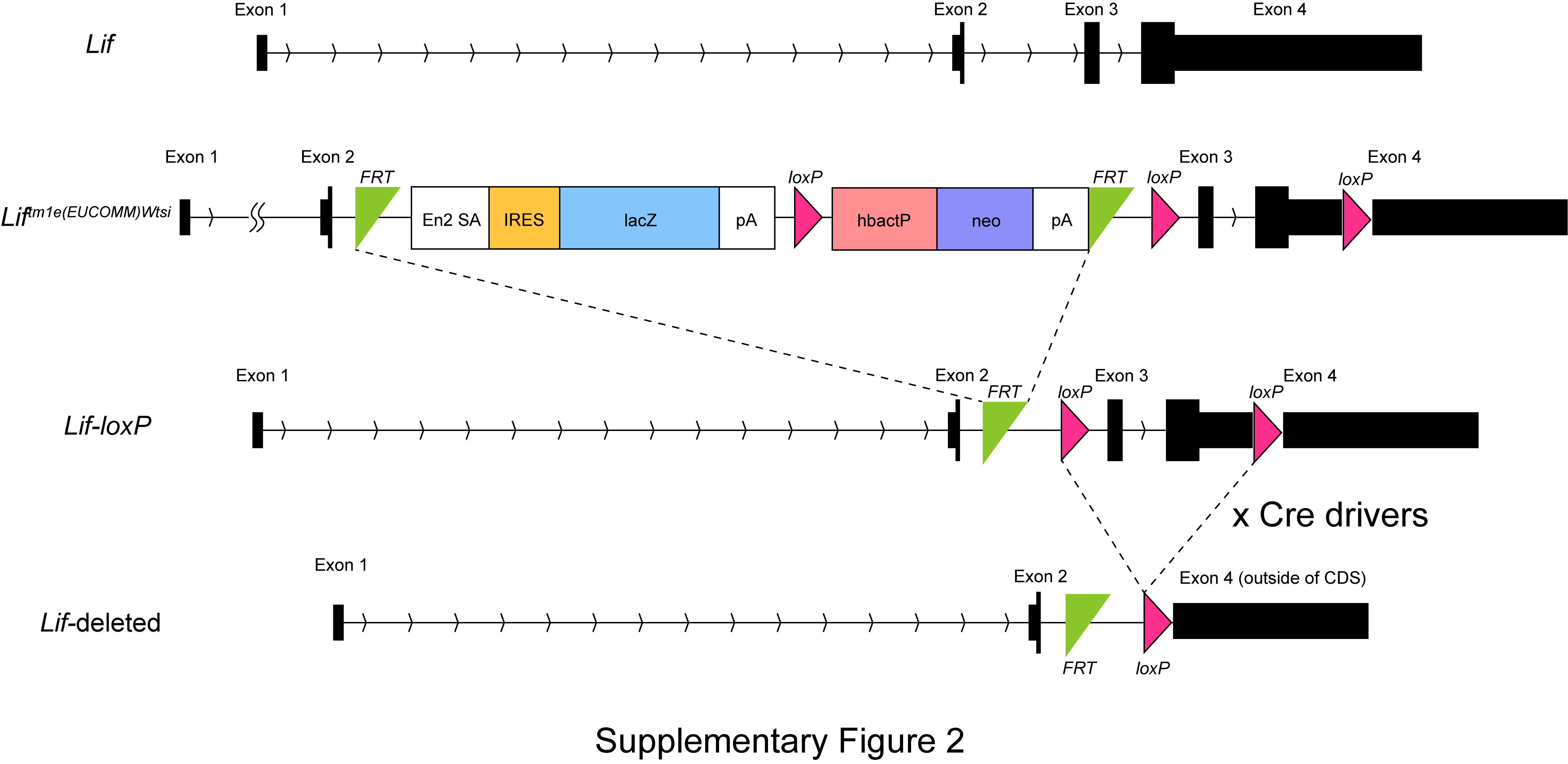


## Supplementary Figure 2. Schematic diagram of the *Lif-*floxed and conditional deletion system.

*Lif* exon 3 and the open reading frame region of exon 4 are flanked by loxP sites. An L1L2_Bact _P cassette flanked by FRT sites was inserted between exons 3 and 4. The FRT-flanked site was removed by mating mice with CAG-Flpo one.


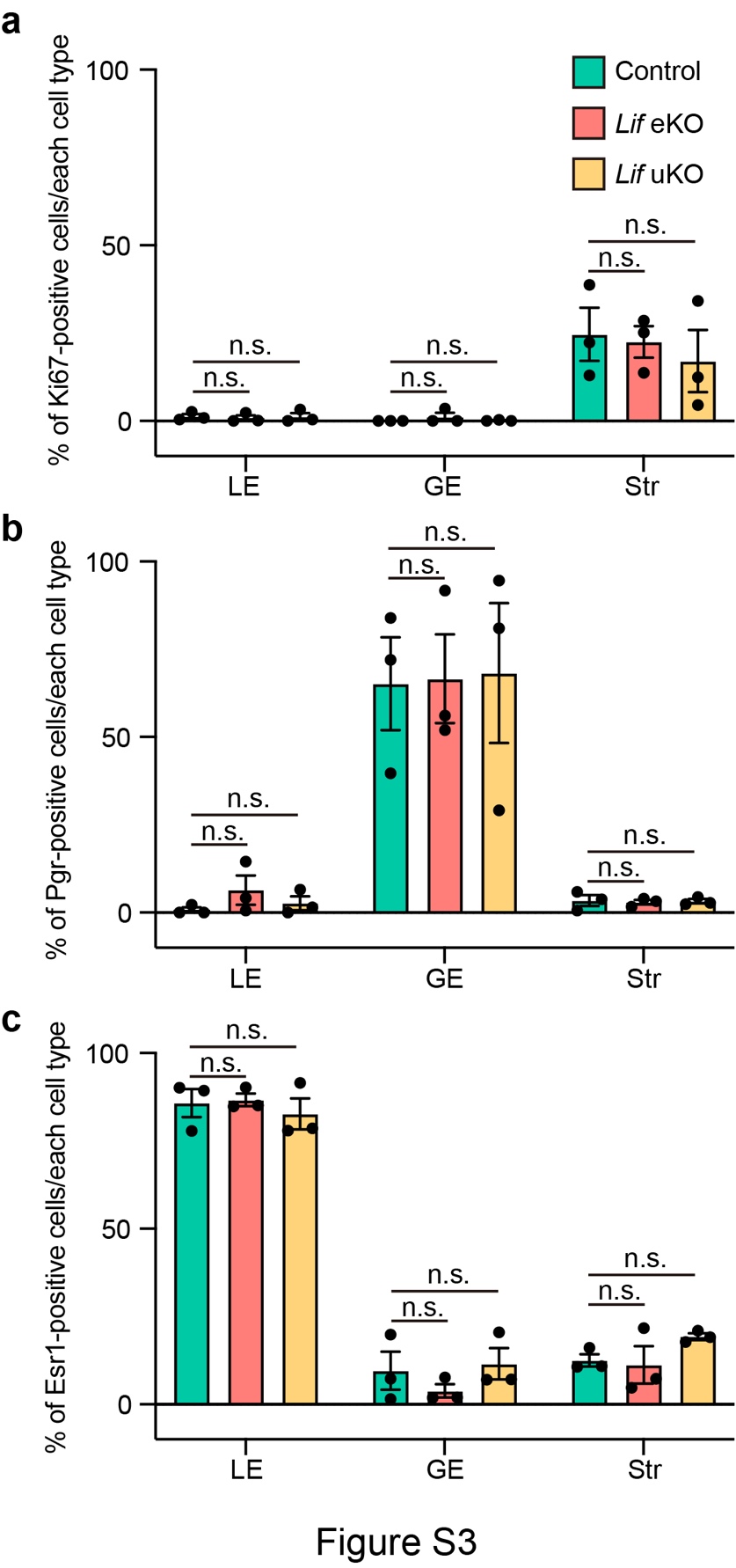


## Supplementary Figure 3. Quantifications of immunofluorescence images in Figure 2.

Quantification of immunostaining signaling of Ki67 (**a**), Pgr (**b**), and Esr1 (**c**) per each cell type was performed. Data are presented as mean ± SEM. n.s.: not significant using two-way ANOVA followed by Bonferroni’s post-hoc test. n = 3 for each genotype.

# Supplementary Movie Legends

**Supplementary Movie1 (separate file).** A 3D view of control pregnant uteri on day 6. See also Figure 5b.

**Supplementary Movie 2 (separate file).** A 3D view of *Lif* eKO pregnant uteri on day 6. See also Figure 5b.

**Supplementary Movie 3 (separate file).** A 3D view of *Lif* uKO pregnant uteri on day 6. See also Figure 5b.

**Supplementary Movie 4 (separate file).** A 3D view of *Stat3* eKO pregnant uteri on day 6. See also Figure 5b.

**Supplementary Movie 5 (separate file).** A 3D view of pregnant uteri on day 6, collected from rLif-treated *Lif* eKO. See also Figure 5b.

**Supplementary Movie 6 (separate file).** A 3D view of pregnant uteri on day 6, collected from rLif-treated *Lif* uKO. See also Figure 5b.

**Supplementary Movie 7 (separate file).** A 3D view of pregnant uteri on day 6, collected from rLif-treated *Stat3* eKO. See also Figure 5b.
